# Learning from Teaching Graduate Students How to Design Climate Change Education Programs

**DOI:** 10.1007/978-3-030-57927-2_7

**Published:** 2020-12-04

**Authors:** Fernando M. Reimers

**Affiliations:** Harvard Graduate School of Education, Harvard University, Cambridge, MA USA; Harvard Graduate School of Education, Harvard University, Cambridge, MA USA

## Abstract

This chapter discusses lessons learned engaging my graduate students in education policy analysis at the Harvard Graduate School of Education in designing climate change education curricula in partnership with educational institutions around the world. Studying those programs developed by my students, I draw out seven cross-cutting themes about what such an approach yields for students, for the educational institutions they partnered with and for my own institution, while drawing parallels between those curricula and the graduate course in comparative education policy analysis in which these curricula were developed. In addressing those themes the chapter revisits some of the central arguments presented in the introductory chapter about the urgency and the challenges of enhancing the effectiveness of climate change education, and some of the key conclusions of critical reviews of the literature on education and climate change about the limitation of existing approaches to the subject.

Those themes are:

- Educating students to address climate change is about engaging them in active problem solving, not contemplation.
- While learning from doing is valuable, to advance the field of climate change education, it is necessary to conceptualize and theorize practice.
- The need to think broadly about learning outcomes in climate change education
- The power of contextually situated learning
- A Signature project-based pedagogy to Change Climate through Education
- Augmenting the capacity for climate change education among teachers and schools
- The limitations of infusing climate change education in existing courses

The chapter concludes examining some blind spots in the climate change curricula presented in the book and drawing parallels between the education response to the COVID-19 Pandemic of 2020 and the education response to Climate Change.

## Introduction

In this book I have presented the approach I developed to teaching climate change education in a professional school of education which consisted of engaging students in projects with educational institutions outside the academy, schools and non-formal education institutions, to assist those institutions in advancing efforts in climate change education. In a nutshell, this approach provides students an immersive experience of learning to do climate change education by doing it in a real world context infusing a project based experience into an existing course on policy analysis.

This pedagogical approach to teach climate change education produces three results: (a) an expansion in the capacity of schools and non-formal education institutions to develop climate change education programs appropriate to their context, as a result of the assistance provided by graduate students and reflected in the programs presented in this book, (b) university students who gain the skills to advance climate change education, and (c) an increase in the body of academic knowledge about climate change education as university students and faculty conceptualize and theorize this practice based work, as this book does. I think of these three products of this interlocking approach to climate change education as akin to the mathematically and nature inspired interlocking paintings of Dutch graphic artist Maurits Cornelis Escher (see https://mcescher.com/).

Notice that the approach differs from a direct instruction, didactic, approach of instructing students on the topic of climate change education, either into an existing course or in creating a course uniquely dedicated to the topic. Chapter 10.1007/978-3-030-57927-2_6 in this book, for instance, is an example of a course uniquely dedicated to the topic of climate change education. The latter has the obvious benefit of allowing greater opportunities to cover more relevant content but the inherent limitations that it requires dedicated faculty and students to the study of the topic, which may limit the number of students who enroll and the number of institutions who adopt the approach. The approach I have developed, which I see as complementary to courses focused exclusively on the topic of climate change which universities may also offer, is meant to make lower resource demands as a way to make it potentially more scalable across schools of education in universities around the world. To use an analogy to the sustainability curricula I developed for primary and secondary schools, which I discuss in the first chapter of this book, a course exclusively dedicated to climate change education would be the equivalent of the ‘world course’, whereas the approach I have proposed here, to infuse into an existing course on policy analysis an option that allows students to learn about climate change education as they partner with educational institutions is analogous to the second curriculum resource I developed in ‘Empowering students to improve the world in sixty lessons’ in which I created an approach to infuse sustainability curriculum widely throughout the entire school, including existing subjects, and offered five lessons per grade to be infused in those subjects. Given the much wider adoption of the ‘sixty lessons’ curriculum, I thought it reasonable to expect the same results in designing a similar approach for the tertiary education level.

The reason I see an approach that makes lower resource demands on institutions of higher education as necessary is because one of the challenges of advancing climate change education, as discussed in Chap. 10.1007/978-3-030-57927-2_1, is the challenge of doing this at scale. Climate change is progressing too rapidly to afford us the luxury of addressing it with small scale approaches to educating people in effective ways to engage them in adaptation, mitigation and reversal. I don’t doubt the merits to have a small group of truly expert individuals, but experts alone are not enough to help either polar bears or humans survive the ravaging effects of climate change. We need to democratize access to effective climate change education and boutique solutions are inadequate to provide the scale such democratization demands. Frankly, this is the reason I underscore the urgency of infusing climate change education in primary and secondary schools, rather than stick only with universities as universities educate directly only a very small share of the world population, whereas most people now have access to compulsory education. This need to find solutions at scale is also the reason I have developed an approach which relies on university-school partnerships as they could become the largest and most widely distributed network of institutions globally to deliver climate change education. Collectively, universities have greater institutional capacity than any other institution I can envision could be deployed to support climate change education, and networked with primary and secondary schools, they can form an ecosystem of incomparable reach and effectiveness. The approach I offer is a path to the development of such an ecosystem.

There is an additional benefit of university school partnerships to advance climate change education, because universities are inherently more cosmopolitan than other education institutions, they can permit the collaboration among people in different geographies, from different walks of life, in this way enabling those participating to recognize the ethical considerations involved in determining burden sharing across generations, across countries and across groups with different levels of privilege.

In this chapter, I draw out seven themes from the analysis of the five curricula created by my students on education for climate change, and from reflection on the approach I created to introduce climate change education in the graduate course on education policy analysis in which these curricula were developed.

## Educating to Address Climate Change Is About Active Problem Solving, Not Contemplation

In engaging with institutional partners to identify the way in which climate change is impacting specific communities and to develop ways to adapt to and mitigate climate change, my then students, and now former students and contributors to this book, learned not just to understand how climate change was impacting these communities. They also underwent a process of looking for various points of entry to make a difference, to help educate others on these issues. This way to learn about climate change education by doing, engaging in actual efforts to address climate change through education, provides richer opportunities to gain understanding of how climate change impacts communities that more detached and uninvolved approaches to study. I contrast this approach to learning about climate change education from engaging in the work from an approach I could have taken, I could have had the students read some of the reports of the International Panel on Climate Change and then, based on that reading, asked them to design a generic curriculum from the comfort of the Gutman Library, the library at the Harvard Graduate School of Education. This approach might have produced a valuable curriculum, and spared my students the additional time and complexities of having to negotiate a curriculum with existing institutions, but it would have been a generic product, specific to no particular place or population. Perhaps some institutions would have eventually found such curriculum useful, or they might have found it, as they did with my ‘World Course’ too detached from their particular goals, resources and constraints. I believe engaging my students first in understanding how climate change was affecting a particular place, and then in discerning how best to educate for climate change in dialogue with local actors, provided them a deeper learning opportunity, making the knowledge gained from the experience more personal than reading a report would have been and teaching them about the particulars of how climate change education can actually be advanced within the constrains and resources available to institutions, than designing a curriculum in a university library without the benefit of such interactions with the messiness of the real world.

The most obvious benefit of this pedagogy of learning by doing is that it can teach the students that the large, and seemingly intractable challenge of climate change writ large, can be tackled by translating it into smaller challenges, located in place and time, identifying specific populations, institutional partners, to address the challenge. In a nutshell, they learned to tackle a large and complex challenge by breaking it into smaller chunks. Because these students did this work not in isolation, but as part of a community of fellow students, which extended into ongoing collaborative work for an additional entire semester after the completion of the semester course in which they began this work, this helped them see their work as part of larger efforts, and made visible how the sum of small actions begins to aggregate into a larger, more impactful effort. Perhaps in the small collegiate community that collaborated in this book they will see a microcosm of the larger global movement necessary to address climate change. Most importantly, I hope this work that they produced, first in designing curricula and then in theorizing their work and communicating it to the scholarly and practice communities in climate change education, will help them see themselves as actors in that movement.

I realize that the curricula my students designed represent relatively small steps, in terms of the many actions necessary to produce systemic changes in the norms and institutions that undergird climate change or that produce sustainable development. Small steps, however, are necessary to help people find pathways that bring about more sustainable futures which will give them hope that change is possible. As Ojala has shown, too many people with knowledge about climate change are emotionally disengaged from the issue, overwhelmed by it, so it is essential to educate in a way that cultivates the right balance between worry and hope (Ojala 2012, 2016). What is learned from these small steps can provide the foundation for more complex understanding, and the efficacy gained from successfully completing these small steps can provide the confidence and the hope, to undertake more ambitious goals in the future. It is too easy for anyone contemplating the sheer complexity of the climate change challenge to drown in the complexity, to become paralyzed and hopeless. The pedagogical challenge, therefore, is to teach students that they can make a difference, and providing an experience at the right level of complexity, is a way to build their efficacy as well as their hope.

Mirroring what they had experienced, the same principle of learning not from contemplation but from action is expressed in the curricula designed by my students, most of which are project based efforts that propose to engage students in active efforts to address climate change in their communities.

One of the benefits of an action-oriented and action-based education approach to climate change is that it enhances the relevancy of what is learned. Learning to design climate change curriculum with strong connections to institutions in which such curriculum will be implemented has valuable self-correcting qualities that tame the risks of running too far afield in the imaginary worlds that are possible in ivory towers. In fact, reflecting on the deficiencies of many of the approaches to climate change education advocated by international development organizations I wonder whether they would have been more successful if those approaches had been developed with the benefit of strong connections to schools that had actually attempted to teach those curricula, and with subsequent opportunities for continuous improvement. Too many of the pronouncements of international organizations on what schools and educational institutions could do to advance climate change education reflect a very limited understanding of how schools and universities actually work and change.

The sheer choice of point of entry in the five projects that my students created illustrates the value of grounding learning about climate change education in engagement with practice: two of them focused on formal education institutions, two on non-formal environments and one on the potential role of schools of education supporting ‘whole of system’ change. This distribution of the foci of these projects resulted from the initial step all students had to follow of considering several alternative ways to address climate change through education in the particular location which was the focus of their work. In that process they considered formal and non-formal education, serving different student populations, establishing different partnerships. Their final choice was deliberate, and the product of analytic reasoning they had to articulate in the final paper they wrote for the course. It was part of the process of policy analysis they were learning in the course as they had to identify how specific impacts of climate change in that locality affected various human populations and to identify which of these populations had to be educated on climate change. As they focused on a particular population, students then had to identify the best pathway to reach that particular population in that jurisdiction. As a result, the choice of whether to focus on schools or non-formal institutions was not an arbitrary choice, made from the comfort of a library, but a choice resulting from engaging in an analysis of the context of practice in which they were grounding their evolving understanding of how education can support people in adapting to or mitigating the impact of climate change.

The resulting three approaches resulting from grounding the choice about these projects in practice differ from the distribution of approaches to climate change education reflected in two recent reviews of research in the field, which show a predominance of studies of school-based environmental education programs, with significantly more limited focus on non-formal environments (Jorgenson et al. 2019; Rousell and Cutter-Mackenzie-Knowles 2020), and an almost nil emphasis on teacher education institutions. A recent review of 221 studies of climate change education concludes, “Given the predominance of research in science education and environmental education, we were surprised to find the literature on teacher education to be relatively limited. Most teacher education studies we did locate focused on the climate change knowledge of pre-service teachers.” (Rousell and Cutter-Mackenzie-Knowles 2020, p. 200). This lack of attention to non-formal environments and teacher education institutions are blind spots of the field, as effective education to address climate change requires more than work in schools and more than curriculum. This demonstrates that anchoring the design of climate change education in a process of analyzing various possible approaches to address the particular needs and opportunities in a specific context, in dialogue with local actors, produces a richer, more diverse, and perhaps more realistic, set of approaches than a top down approach that begins in the university library or even in the offices of development professionals of international organizations. This discipline of learning to collaborate with practitioners is valuable to help develop a perspective on the relevance of academic knowledge. A critique of research on environmental education argues that an important criterion to assess such scholarship is to ask whose ignorance does research reduce, proposing that too often the ignorance of researchers is not well aligned with the ignorance of potential users of the knowledge generated by research (Gough 2002, p. 19).

## While Learning from Doing Is Valuable, to Advance the Field of Climate Change Education, it Is Necessary to Also Conceptualize and Theorize Practice

Two purposes are advanced as students conceptualize what they learned by doing as they engaged in a practice of designing a climate change education curriculum. The first is that theorizing practice advances the field of climate change education. The second is that conceptualizing practice contributes to professional preparation.

Given the contested nature of the field of climate change education, particularly whether it is a distinct field from environmental education and education for sustainable development, more research and theorizing are necessary to develop the knowledge base that would help the field mature. I argued in the introductory chapter of this book that there are two reasons to engage university faculty and students in the enterprise of supporting climate change education at precollegiate levels and in non-formal environments. The first is so that they can support the necessary development of capacity that the enterprise demands. The second is that universities can experiment, evaluate, conceptualize and theorize a field where, at present, practice leads theory, in order to transform it into a distinct field of practice guided by expert knowledge and supported by the powerful tools of logic and science.

For this reason, the engagement of students with a context of practice, and their work developing a climate change education curriculum, was only half of the work they had to complete in my course on education policy analysis. This engagement in practice had to be complemented by an analysis of their work, translating the knowledge they had gained into public knowledge, receiving feedback to their ideas in a conference and from academic peer reviewers, and finally publishing their work so it would become open to academic scrutiny and debate. In effect, the students engaged in a two-step of ‘translation’, first translating research in the field using it to inform the curricula of climate change education they created, and then translating back what they had learned from creating these curricula into academic scholarship which entered the academic conversation. Their chapters in this book reflect that second aspect of the work. If universities embraced the challenge of engaging more students, as part of their education, in similar activities developing approaches that can guide climate change education, we could accelerate the development of this field considerably.

Learning to conceptualize professional practice is also good professional preparation in general. In his book, *The Reflective Practitioner,* MIT Professor Donald Schon (1983), explained how one of the essential elements of professional preparation is to equip students with the skills and tools to reflect on their practice, and to draw from their practice knowledge which they can then make publicly available, falsifiable, supporting further professional practice as well as further research and scholarship. This ability to reflect on the knowledge which guides practice is essential to the improvement of professional practice. Practitioners often guide their practice with knowledge to solve problems that goes beyond the mechanic application of principles or conclusions drawn from academic knowledge. Schon also argued that the failure to comprehend this all too often leads institutions involved in professional education to base the curriculum on a paradigm which assumes that professional practice is simply the application to problems of practice of the general principles drawn from academic research in the fields associated with that practice.

Schon’s epistemological stance recognizes that when practitioners solve problems they learn from the consequences of their actions, and the knowledge they gain makes them better at solving problems in the future, hence better professionals. Solving problems, especially complex, messy, adaptive or divergent problems such as climate change, is thus much more than mechanically applying lessons drawn from research to new situations, and involves forms of creation, design of solutions and experimentation. While good professionals learn from these ‘private’ experiments that constitute their practice, this knowledge is often accessible only to the practitioner, because it is not processed in a way that allows others to learn from it or to falsify it. This is called ‘tacit’ knowledge. Constructing opportunities to learn from such knowledge, making ‘tacit’ knowledge ‘public’ is thus critical to professional education, and to the advancement of the profession.

Some of the critiques to professional education based in universities, concern whether the curriculum provides sufficient access to knowledge essential for effective practice, or whether it is too ‘theoretical’ too ‘disconnected’ from the fields of practice for which they are preparing people for professional practice. In *The Reflective Practitioner* Schon argues that the classical worldview that sees practice as a mere application of foundational principles is responsible for this disconnect. The approach I developed, which engages students first in designing an educational program in dialogue with partners in institutions of practice, and then in examining the results of their work and discussing it in the context of the larger academic scholarship on climate change education attempts to close the disconnect described by Schon.

## What Outcomes Matter in Climate Change Education

The key learning outcomes that I sought to help my students gain were the ability to understand the interrelations between climate change and specific populations, to identify knowledge, attitudes and skills that would help those populations adapt to and mitigate climate change, to develop an actionable approach to climate change education in those communities that would help those population gain such competencies, anchored in a robust theory of action and in a feasible strategy of implementation, with adequate local support to ensure sustainability. I further sought to help them reflect on what they had learned, and conceptualize and communicate their efforts in ways which made the knowledge they had gained visible and public, so that their experiments represented a contribution to advancing the field of climate change education. Knowing a topic, no matter at what level of depth, is only one component of the competency to deploy this knowledge in service of transformative goals, such as supporting school change.

In a nutshell, I sought to help my students break down a complex problem such as how to educate about climate change, into a tractable, smaller, problem, and to design a solution that could be tested in a particular social context. In this way, I hoped to help them become aware of their own power to make a difference, individually and collectively, in changing systems that undergird climate change education. The power and the desire to personally engage, the competency to address climate change education, requires more than knowledge of the research on climate change education or the research on climate change. It requires the cultivation of self-knowledge, of ethics, of empathy, or hope, or skills to collaborate with others. That breadth of skills is what a project based immersive experience in having to solve a real problem of practice helps develop in ways which simply reading reports in the library about climate change or research on climate change education do not.

So far, the designs my students have produced, and these chapters, are testimony of what they learned from this experiment. The impact of this book in the field, and my former students eventual continued engagement with the efforts they began in this course and with climate change education more generally, will provide evidence in years to come to evaluate some of my hypotheses on the long term outcomes of this form of professional preparation.

Specifically, I sought to help my students gain the skills to develop a context specific strategy for climate change which addressed these questions:

1. What are the specific impacts of climate change in this jurisdiction? How do they impact various human populations? Which of these populations needs to be educated on climate change?
2. What knowledge, dispositions and behaviors could mitigate the impact of climate change and are there ways in which changes in the behaviors of populations in this jurisdiction could slow down climate change?
3. What are the means of delivery to reach each of the specific populations in this jurisdiction?
4. What curriculum can best educate each population?
5. What institutions can support the development of the institutional capacity necessary to deliver such curriculum effectively?
6. What institutional collaborations can support the implementation of this strategy?

From this analysis, I sought to help my students develop and assess climate change education programs grounded in sound logic theories, where they could make explicit the hypotheses which undergird any climate change curriculum or instructional program. I also sought to help them identify and assess, weighing various criteria, alternative paths to address the root causes that undergird the lack of knowledge, skills and attitudes that prevented the populations they were serving from adapting or mitigating climate change.

The chapters in this book demonstrate that students now have those skills. In these chapters, my former students demonstrate that they competently used policy analysis approaches to assess alternatives to climate change education, and to develop a sound logic theory to sustain their program, addressing the six core questions.

I see less evidence, however, that I succeeded in helping my students construe the implementation of such a curriculum as an adaptive challenge that would require attending to the cultural, psychological, professional, institutional and political dimensions of the enterprise (Reimers 2020).

Relatedly, I see only incipient evidence that the curricula designed by my students aim to help those who engage with that curricula develop an understanding of the systems that undergird climate change. This emphasis in understanding systems is most evident in Chaps. 10.1007/978-3-030-57927-2_2 and 10.1007/978-3-030-57927-2_3, the school-based curricula in the Middle East and Guatemala. I was aware that the opportunity to gain the skills to understand and transform systems eludes most of the efforts of climate change education examined in a recent review of research (Jorgenson et al. 2019) and had hoped that grounding an approach to climate change education in engaging students with local institutions and local actors, would seamlessly translate into understanding the systems which undergird current climate education challenges, and to discern what kind of collective action would be involved in changing them. I assumed that such engagement would translate in understanding changing education institutions as an adaptive challenge requiring a multi-stakeholder coalition that could produce collective leadership to sustain the change effort.

Perhaps the reason I did not achieve this goal stems from the inherent limitations of conducting this work within the timeframe of an academic semester in the context of one of the multiple courses students are taking. It is possible that a more intense immersive experience, for instance working solely on this project for an entire semester, and using that work as the anchor for inter-disciplinary integration of all other academic study in their graduate program, might have produce deeper learning. A different engagement, in which students could sustain partnerships with schools through various cycles of implementation, revision and adaptation of the curriculum, might also have made more visible to them the ‘systems’ which have to be changed in order to institutionalize their climate change curriculum. While I hope that they will continue such engagement with the institutions that they partnered with, that work will now take place outside the context of the course. It is also possible that I should have relied on more didactic approaches to teach some of this knowledges, rather than expect that it would be an inevitable by-product of the experiential learning I was engaging my students in. There are other limitations resulting from carrying out this kind of action based climate change education during a semester long course, we overcame them in part because all students continued to work on these projects after the semester had ended, particularly making revision to their chapters and responding to the various rounds of feedback they received. While it is satisfying to know that there is enough interest in learning for its own sake so that a group of dedicated students can commit to work an entire additional semester, after they had completed the course on which this work began, on a project of this sort when this does not fulfill any requirement or has any grade attached to it, I wonder if this approach is scalable. An optimistic interpretation is that if a learning experience truly engages students, that engagement can sustain continued learning even when the original structures that supported it, the course requirements and the academic credit, are no longer there.

It is also possible that there are limitations to how much can be expected from an infusion approach to helping students learn about climate change education in the context of existing courses. As I explained earlier in this chapter, these limitations of the approach I followed are also its virtues. While the engagement in practice this semester-long, turned into year-long, assignment provided students was somewhat limited, perhaps not sufficiently intense, or long or immersive, that is also why I was able to fit it into my course, and students were able to fit it into their schedules which included other courses in addition to the one they were taking with me. If I compare the approach I developed and implemented in my course with the K-12 sustainability curricula I had developed, what I did was more similar to the 60 lessons curriculum than to the original ‘world course’. Recall that the ‘world course’ was an intense and immersive experience, engaging students in a deep, coherent and rigorous sequence of 350 units, requiring 10 h a week from kindergarten to high school. This was, perhaps, too much of a good thing, too demanding for most teachers to be able to implement in their schools. Translating this ‘heavy’ approach to sustainability education into a ‘lighter’ 60 lessons curriculum, five lessons per grade, made the approach a lot more usable and scalable. My hope is that the approach I have developed and describe in this book, to create opportunities to develop climate change education curricula infusing them into existing courses, is more scalable, than proposing an immersive climate change education course, the parallel to the ‘world course’, that students should take. Certainly, students may learn more about climate change in that dedicated course, but it is likely that such an approach would reach fewer students than infusing climate change related assignments in a wider array of existing courses.

Similar to my learning goals for my students, the learning goals of the curricula which they designed espoused similarly ambitious goals for the students they sought to serve. Chapter 10.1007/978-3-030-57927-2_2, the curriculum to cultivate climate change leaders in the Middle East, seeks to foster leaders who can engage in civic action around climate change, and who are capable of practicing empathy, systems-thinking, media literacy and collaboration. The curriculum that Margaret Wang and David Rhodes designed included not only an essential foundation in the sciences, but critical skills to interpret scientific information, along with agency gained in a hands-on project that teach students the role of complex power structures, systems and the needs and interests of various stakeholders. The curriculum aimed at teaching students how to engage with climate change as individuals but also collectively to affect climate change.

Chapter 10.1007/978-3-030-57927-2_3, the curriculum of whole school climate change in Guatemala focuses on helping students learn to think about systems, understand climate change as a shared responsibility in order to change systems.

Focusing on marginalized populations, Chaps. 10.1007/978-3-030-57927-2_4 and 10.1007/978-3-030-57927-2_5 focus on non-formal education approaches. Chapter 10.1007/978-3-030-57927-2_4 focuses specifically on how to mitigate the impact of climate change among vulnerable populations in Haiti using radio education to develop knowledge, behavior and skills that minimize the harm caused by climate-related disasters and to help them adopt sustainable agricultural methods. Chapter 10.1007/978-3-030-57927-2_5 focuses on out of school youth in Pakistan to develop social and collaborative skills that help them work in teams and develop their own solutions to mitigate the impact of climate change in their lives and to discover their voice in advocating for their rights. In Natasha Japanwala’s words: “it is important to teach students how to advocate for themselves — not just so that they can demand better resources and negotiate if they have to migrate, but because they should be able to conduct public protests or speak to local authorities if need be.”

Chapter 10.1007/978-3-030-57927-2_6, in proposing student led curriculum about climate change in schools of education, advocates tapping the power of students to transform the contexts in which they are learning, essentially cultivating their agency and efficacy in transforming systems as a way to contribute to a grassroots movement of climate change advocates.

## The Power of Contextually Situated Learning

The pedagogy I followed in teaching my students about climate change invited them to find a specific context on which to ground the practice they would engage through the course and beyond. I actively discouraged students from writing ‘generic’ guides or curriculum of climate change education, and instead urged them to identify a specific site and institutional partners so their practice could be constructed in dialogue with those partners. My main goal was to ground this work on dialogue with local actors that would cultivate deep empathy with specific communities and institutions, and help my students learn how local populations articulated the impact of climate on their lives and the role of education in helping address this impact. This helped students investigate the specific ways in which climate change was impacting particular communities, and from there, discern what kind of learning outcomes would be of greatest value to those particular communities. This crucial step is, by necessity, missing from any generic guide on education about climate change designed to serve a multiplicity of contexts.

Predictably, once my students focused on the specific learning outcomes that would be of value to the students participating in the programs they were designing to cope with or mitigate climate change, the education programs they designed focused also on those specific manifestations of climate change. The leadership curriculum presented in Chap. 10.1007/978-3-030-57927-2_2, for instance, engages students in observing environmental degradation in their particular communities, and in developing causal models and networks of persons or institutions implicated in the process, using this to teach students systems thinking.

In designing a whole school approach to climate change for Guatemala, Chap. 10.1007/978-3-030-57927-2_3 analyzes both the limitations of UNESCO’s generic guide of whole school change, as well as the limitations of Guatemala’s curriculum on climate change, developing an approach grounded on the particular strengths and limitations of a particular school. Their analysis of the shortcomings of the national curriculum calls out “the overall lack of interdisciplinarity within the Base National Curriculum, and of teacher professional development opportunities geared towards building competencies on environmental and climate topics, indicate a lack of acknowledgement among the country’s educational authorities about the complex and interdisciplinary nature of this issue.”

Chapter 10.1007/978-3-030-57927-2_4, proposing a radio education program to educate Haitian adults to mitigate the impact of hurricanes in their lives, recognizes the shortcomings of the formal education system in excluding many students and in leaving many graduates very poorly educated. It is this analysis of context which leads the authors to focus on adult education as an essential avenue to address the impact of climate change on vulnerable communities.

Similarly, Chap. 10.1007/978-3-030-57927-2_5 recognizes that addressing the impact of climate change on the most vulnerable requires focusing on those who are poorly served or excluded from the formal education system. Focusing on a particular population of out of school vulnerable youth in southern Pakistan, the authors of the chapter integrate a climate change education curriculum within a life skills and literacy skills curriculum. This contextual grounding guides also the very content and structure of the curriculum:

The first step of the curriculum is helping students understand what climate change actually is, rather than instructing them about climate change as it is occurring around the globe, the introductory unit of the curriculum focuses on three phenomena that impact Badin directly and that the students will have observed around them: overheating in the summer, floods because of sea levels rising, and the lack of productivity of the land because of salinization. This is largely in line with the overarching philosophy driving climate change curricula: for example, the US-based National Center for Science Education (2016) describes four best practices for climate change education that can be considered universal: “Make it local, make it human, make it pervasive, make it hopeful.”

A distinctive feature resulting from grounding these projects in specific contexts visible in the two programs designed for formal schools is that they are integrated with existing curriculum in use in those institutions. The leadership curriculum in the Middle East builds on an analysis of ongoing curriculum efforts to teach about the environment and climate, and relates the proposed curriculum to them. The whole school approach to climate change proposed for Guatemala is also anchored in an analysis of the current curriculum in Guatemala.

Chapter 10.1007/978-3-030-57927-2_6 illustrates the power and the limitations of contextually situated learning. The goal of the chapter is to formulate an approach that can support education for climate change by educating broadly students that prepare for various professional roles in education, the ‘proof of concept’ is developed in the specific context of the Harvard Graduate School of Education. While there are similarities across schools of education, there are also particularities in how they are governed, for example in how open they would be to having students propose and lead a course on climate change. This inherent limitation of developing a case deeply rooted in a unique context is shared by the other projects presented in this book.

Another benefit of grounding this pedagogy of learning to address climate change in local contexts is that it helped students engage with local actors, and develop the skills for collaboration that will arguably prepare them to continue to advance the necessary systemic change to curb climate change.

## A Pedagogy to Change Climate Through Education

In my course on education policy analysis I adopted a problem- and project-based pedagogy designed to cultivate the agency and problem-solving skills of my students engaging them in co-constructing the curriculum they were experiencing. Essentially, I offered four alternative paths to develop the skills in policy analysis which were the subject of the course, one of these paths was designing a climate change curriculum. I then offered guidance on how to find a context and which questions to address, meeting with those teams as necessary, but for the most part letting them do this work independently. The support the students received came in the form of readings such as the reports of the International Panel on Climate Change and some existing curricula on climate change and readings on climate change education, sharing my own experience designing sustainability curriculum and working with schools and school networks around the word. The students were supported more generally in the course with other readings on system level change, curriculum and teacher education, that were the basis of lectures, classes and discussions in this course, but those focused on comparative education, not on climate change education in particular. Students did receive feedback to various papers they wrote, all of them building up to the final paper in the course, and of course they had the benefit of a community of peers working on similar topics. But the intellectual autonomy and freedom that undergird my pedagogy was closer to that which is typical of writing a research paper, or a dissertation, than that which is typical of a scripted graduate course in which students have to complete pre-specified assignments or tests which are then graded on a rubric.

Unsurprisingly, but to my pleasure, my students followed similar pedagogical principles in the curricula which they designed. Whether this is because this assignment in my course attracted students with a penchant for intellectual autonomy, or because experiencing it caused them to seek similar freedom for the students they were serving is something I am not able to determine. Chapter 10.1007/978-3-030-57927-2_2 proposes a student centered, project-based curriculum in the Middle East, as does Chap. 10.1007/978-3-030-57927-2_5 in Pakistan. Chapter 10.1007/978-3-030-57927-2_6 takes this idea even further, by proposing not a student co-constructed curriculum, but a student led, and faculty supported curriculum in the very same institution in which I teach. Only the adult education curriculum via radio-education in Haiti adopts a didactic approach with a specific focus on helping adults develop knowledge, attitudes and skills that can help them minimize the impact of hurricanes on their lives. The emphasis adopted by most students on project-based pedagogies resonates with the conclusion of a recent review of climate change education programs, which identifies a gap in the existing literature in terms of approaches to climate change education which are participatory, interdisciplinary, creative, and affect-driven (Rousell and Cutter-Mackenzie-Knowles 2020, p. 191).

We found that the four approaches which have dominated the literature on climate change education were generally top-down approaches, whether the focus was on scientific knowledge, formal curriculum, behaviour change, or mitigation/adaptation. Yet underneath this entrenched edifice of top-down education and disaster management, a series of innovative, bottom-up approaches have begun to emerge. These include participatory approaches which empower communities of learners to design their own climate change projects and modes of engagement with the issue. (Rousell and Cutter-Mackenzie-Knowles 2020, p. 202).

More specifically, this review identifies a pressing need for research that gives young people both a hand and a voice in redressing the complex implications of climate change in their own communities and environments. Our analysis calls for new ways of making climate change meaningful for children and young people through participatory and arts-based modes of engagement. In effect this is extending climate change education and its research beyond the realms of understanding young people’s climate change science knowledge (or lack thereof), which has no bearing on climate change itself, to far more important and pressing aims which actively empower children and young people to mitigate climate change. (Rousell and Cutter-Mackenzie-Knowles 2020, p. 203).

The non-formal education curriculum for out of school youth in Pakistan articulates the importance of this type of pedagogy to the critical outcomes it seeks to develop. As Natasha Japanwala described,

Using design thinking and project-based learning to teach adaptation strategies has value beyond familiarizing the students with new skills and the ability to work collaboratively towards a common goal. It builds their leadership skills by encouraging them to think of themselves as climate change ambassadors in their community, especially since the exercises follow a unit where their conceptual knowledge was built. They have, through units 1 and 2, had both the experience of leadership and the vocabulary to articulate the cause they are leading.

The curriculum I developed was a climate change education strand embedded in a comparative education policy course in which students would work on projects to support schools or other education organizations, one of four options students could pursue to practice their policy analysis skills. As such, my course was not interdisciplinary but very much a course on education policy and comparative education – although comparative education is an interdisciplinary field. We drew on knowledge on teaching and learning, education system change, and policy analysis. Obviously additional knowledge informed the work of my students, but this was knowledge I assumed they would bring in, or gain independently as they worked on their projects. In other words, I thought of the action projects in which the course engaged them as the occasion that would help students generate questions which would lead them to access the bodies of knowledge necessary to solve the problem. This was especially the case in investigating the way in which climate would affect the particular populations and localities of their projects, an area on which I played no role in instructing them, other than suggesting resources they could consult. All learning in this area was self-initiated and self-directed.

This is one area in which the curricula my students designed does not mirror the curriculum they experienced in the course. Most of them did propose interdisciplinary approaches. Perhaps this is because there are certain affordances and assumptions one can make in teaching Harvard graduate students which are less feasible when teaching at lower levels of education. In particular, the two curricula designed for the formal education system, in the Middle East and in Guatemala, were interdisciplinary. With respect to the two curricula designed for vulnerable out of school population the curricula were interdisciplinary too, although the implementation vehicle for those curricula was not structured around disciplines and subjects in the same way schools are. The student led curriculum for the Harvard graduate school of education presented in Chap. 10.1007/978-3-030-57927-2_6 is also interdisciplinary, although it benefits from the same affordances my course did, on relying on background knowledge of students and on their ability to learn what they need to work on their personal projects.

## Augmenting the Capacity for Climate Change Education Among Teachers and Schools

One of the explicit aims I sought in urging my students to think about climate change in specific contexts and with concrete institutions in mind was to help them consider institutional capacity in their efforts to address climate change education. As I explain in the introductory chapter of this book, it is the lack of attention to institutional capacity which accounts for the insufficient results of past efforts in climate change education. This theme is also a central tenet of the course on comparative education policy analysis where this work on climate change was carried out. The readings in the course support the proposition that policies are not self-executing and that good policy analysis considers implementation as part of the process of analysis itself, and not as an afterthought. My students’ designs demonstrate that they understand the necessity to think about capacity to implement a climate education program, and that any climate change curriculum must achieve a balance between being within reach of the existing institutional capacity, while creating the opportunities to strengthen that capacity. All chapters in this book identify institutional partnerships as avenues to strengthen the institutional capacity of teachers and schools and propose integrating programs of professional support to help teachers develop their capacity.

Chapter 10.1007/978-3-030-57927-2_2, the leadership curriculum for the Middle East, argues the value of a partnership with a higher education institute, the Arava Institute, as a way to strengthen the capacity of teachers to teach about climate change. It also proposes an iterative process of improvement in which teachers collaborate with others in professional communities as a way to develop their capacity to teach about climate change. What is very clear in the programs presented in this book is that strengthening institutional capacity is not an afterthought to the design of a climate change education program, but an integral part to the design of the program.

Chapter 10.1007/978-3-030-57927-2_3, identifies teacher professional development as a crucial omission in UNESCO’s guide on whole school climate change education (Gibb 2016), and proposes an approach to build that capacity in the school that is the focus of their project through a partnership with a local environmental education ‘Amigos del Lago’.

The two projects focusing on nonformal education include an analysis of possible institutional partners with the necessary capacity to execute the programs.

Chapter 10.1007/978-3-030-57927-2_4, focusing on radio education, maps a network of institutional partners with the know how to design and deliver high quality radio programs.

Chapter 10.1007/978-3-030-57927-2_5, embeds the program of climate change education into the programming of an institution working to develop literacy and life skills among the target population for the program, the Citizen Foundation, and recognizing the financial limitations of the organization, is explicitly designed to be low cost and low resources.

Chapter 10.1007/978-3-030-57927-2_6, adopts an entrepreneurial approach to the question of capacity. Recognizing the limited existing capacity in schools of education to educate for climate change, it proposes to mobilize an underutilized resource, the students, and makes them the agent of curriculum renewal. This potentially transformative idea assumes, of course, the leadership capacity to embrace this role among students.

## Blind Spots

A recent review of research on climate change education argues that addressing climate change education requires more than changes in individual consumption, but collective action that can change systems. That review identifies as a limitation of the field that much of the research, and of the practice, in climate change education is based on a mindset focusing on individual impact on climate change (Jorgenson et al. 2019, p. 160). The authors of the review argue that reducing the risks of climate change requires a sociotechnical transition towards more renewable-energy systems which would call for complex social, technological and ecological changes. In their view, a relevant climate change education needs to help students understand and bring about those changes. At present, the reviewers argue, most environmental educators and researchers are focused on influencing individual behavior, thus privatizing the concept of environmental action.

By minimizing the role of collective action, environmental educators and researchers may be reinforcing a simplistic and narrow conception of the relationship between climate change, human action, and energy system change and distorting the fact that many of the most impactful climate actions are decisions about energy supply systems that are made by state and market sector actors under direct pressure from advocacy coalitions and other social collectives. (Jorgenson et al. 2019, p. 166).

The five projects created by the students in my course follow within the more conventional paradigm focusing on influencing individual behavior rather than on collective action across diverse stakeholders and institutions that can help bring about the sociotechnical transition towards renewable energy systems. While some of the projects, such as the leadership project in the Middle East or the non-formal project in Pakistan, emphasize the role of collaboration and collective action, the scope of that construct is limited to collective action among students or adult learners in the context of school projects or literacy and life-skills programs. It does not contemplate educating for collective action across institutions and diverse stakeholders and lacks explicit connections to macro-level transformations of energy systems. This could be the result of focusing on schools or non-formal education institutions as the point of delivery of the curriculum, as it is a conventional way to see schools as working in some isolation from other institutions. While several of these projects propose partnerships between schools and other institutions, for example the projects in the Middle East and Guatemala, these are generally for the purpose of enhancing the capacity of schools to teach about climate change. There are obvious limitations in the scope of a one semester course to engage students in partnerships with schools that do much more than work within the boundaries that schools accept as legitimate for their work. The notion that more effective environmental education would require creating opportunities for students to collaborate with multi-actor networks, such as climate scientists and activists, renewable energy firms and entrepreneurs, state agencies, NGOs and civic groups, is probably outside the scope of what a group of students can realistically help orchestrate in collaborating schools over the course of a semester.

A seeming logical byproduct of developing climate change education programs which are contextually situated does not seem to have materialized in these five projects. One could expect that any design for specific contexts would take note of existing knowledge and beliefs about climate change. The importance of designing climate change education taking existing beliefs, attitudes and contexts of learners into account, rather than assuming they are blank slates is underscored in a critique on how current approaches to climate change curriculum shape attitudes and behaviors towards climate change (Brownlee et al. 2013). In none of the curricula presented in this book is there a recognition of what those pre-existing beliefs and attitudes are in the specific contexts for which these curricula were developed.

The curricula developed by my students demonstrate only an emerging understanding of the interdependence of climate change with wider processes such as poverty alleviation and sustainable development. While most of these curricula address the impact of climate change specifically on people in poverty, and four of the five are focused on the developing world, there is no specific recognition of the gendered experience of climate change.

Finally, a word about a tension in a project which began with the premise that in order to be more effective, climate change education efforts would need to be well grounded in specific local contexts. As I argue in the introduction of this book, there are limitations to curricula or instructional materials that are written at such level of generality that the ignore everything that is defining of any particular context. This is not to say that every program of climate change education needs to be developed from scratch for every particular context. To make such radical case for the need of idiosyncratic climate change education would be to negate the possibility of building a generalizable corpus of knowledge that can inform the enterprise. I am not making such case. But I am suggesting that it is important to add to the corpus of knowledge that can help make climate change education a distinct field of study and practice, knowledge that is the result of particular efforts to transform practice. Each of those efforts can be considered an experiment of sorts. The reports of such experiments need to be conceptualized so that subsequent reviews can identify generalizable principles from them. We obviously hope, in writing this book, that what we learned in these five settings would be of value to other climate change educators in different settings, and of value to those interested in theorizing the field. I am also hopeful that while this book reports on a pedagogical experiment on climate change education in a single course at the Harvard Graduate School of Education, the analysis offered here would be of value to colleagues in other institutions working in schools of education and perhaps even in other fields such as public health, public policy, government or business. I have offered the premise that if more universities engaged with this work, this would help fill an important gap in institutional capacity that is at the core of the limited effectiveness of present climate change education efforts. This premise can only be tested if a sufficient number of colleagues in other institutions, and even in my own, accepts the invitation to give this a try, and this book is meant to suggest that it is at least worth trying.

## Coda: Writing About the Role of Universities in Climate Change in Education During a Pandemic

I review these concluding lines in mid september of 2020, six months since Massachusetts public health authorities instituted various physical distancing measures to contain the spread of the COVID-19 Pandemic. My course last semester shifted to online instruction on short notice, as did the instruction of most educators around the world. We have recently started the academic semester online, in what will be the first ever entire academic year in which all curriculum will be delivered online at the Harvard Graduate School of Education. Globally, at the time of writing, more than twenty nine million people have been diagnosed as infected and more than 929,000 people have died from COVID-19. I suspect many more will still be infected and die until an effective treatment is available. The prospects of developing and distributing a vaccine to prevent infection or of discovering and effective treatment to mitigate the lethality of infection are at best six to twelve months away. In addition to the direct impacts of the pandemic on health and life, other indirect effects ravage the planet: unemployment, hunger, poverty, rising authoritarianism. I certainly hope this pandemic does not lead also, as did the Flu Pandemic of 1918 in Germany, to the breakdown of democracy and the rise of fascism (Blickle 2020). Underscoring the severity of the crisis created by the Pandemic, Antonio Guterres, Secretary General of the United Nations, has characterized it in this way “The Pandemic is more than a health crisis, a security crisis, and a human rights crisis. It has affected us as individuals, as families and as societies. The crisis has highlighted fragilities within and among nations. It is no exaggeration to suggest that our response will involve remaking the very structures of societies and the ways in which countries cooperate for the common good. Coming out of this crisis will require a whole-of-society, whole-of-government and whole-of-the-world approach driven by compassion and solidarity.” (un.org/en/coronavirus/UN-response) 

I have focused the last six months in assessing the educational needs and the education responses to the pandemic around the world, and developing tools which can help education leaders develop strategies for education continuity. As I was concluding this book during this period of physical distancing and living and working online, I see in this pandemic and in our inadequate education responses to it some parallels to the topic of climate change and our lack of adequate education responses.

In most countries, the education response to COVID-19 was slow, reactive and short term, rather than proactive and long term (Reimers and Schleicher 2020a, b). As a result, there are vast differences across schools and jurisdictions in how children from different circumstances have been impacted by this pandemic. Some have benefited from sustained opportunities to learn, through various means. Others, not so much. As a result, learning gaps are increasing and it is likely, because further periods of physical distancing are to be expected until there is a vaccine to prevent infection or an effective treatment to reduce the health impact of infection, that learning loss resulting from these gaps in learning opportunities will continue. If they do, this pandemic could cause the greatest global loss in opportunity to learn in decades, if not centuries.

Such massive education losses were not the inevitable result of a pandemic, and neither were the number of people who have been infected or who have died. The health impact of the pandemic has been mediated by the institutional capacity and leadership effectiveness in various jurisdictions. At present, the reported number of COVID-19 related deaths per capita varies widely across the world, alongside the preparedness and efficacy of the response of public health and political authorities, and the ways in which the data is recorded and reported. In some jurisdictions, leaders paid attention to the evidence, took the advice of public health authorities, and acted decisively and effectively. In other jurisdictions, not so much. As a result, the number of deaths per 100,000 people ranges from 96 in Peru, 65 in Bolivia, 64 in Chile, 59 in the United States, to 6 in Finland, and even less in India, Poland, Egypt and a number of other nations (Johns Hopkins University 2020). As time goes by, and we obtain more accurate estimates of number of actual deaths related to COVID-19, which are probably underestimated at the moment given deficient diagnostic measures, and as the pandemic runs its course throughout the world, perhaps in several waves of infection, we will have a better sense of the extent of these differences.

Similarly, the education effects of the pandemic are also mediated by the institutional capacity and leadership effectiveness of various jurisdictions, even of different schools. Where there was readiness to sustain education remotely, where there were established alternative delivery mechanisms, such as connectivity and devices to reach students, and where students had skills for independent learning, there has been more educational continuity than where such alternative delivery channels or skills for independent learning were missing.

As a result of the deficient education preparedness and response, as countries have enacted regimes of physical distancing, millions of students around the world are stranded like polar bears as their learning platforms have melted, watching their learning opportunities vanish.

COVID-19 is a high impact event just like climate change, only it is happening much faster. Perhaps it is the sudden and rapid nature of the spread of this highly contagious virus that impeded our capacity to adapt our educational institutions so they could continue sustaining learning under different conditions to help us cope with this massive change to the way we live. However, since the early days when the World Health Organization declared the pandemic, various models projecting the rate of infection of COVID-19 could have helped inform what actions could be put in place to slow down the spread of the virus. Some jurisdictions made better use of that information than others, with consequential results for the lethality of the pandemic across jurisdictions.

Climate change is a high impact event as predictable as a pandemic. The various effects of Climate change can be predicted with a particular likelihood of certainty, and the reports of the International Panel on Climate Change provide those estimates (IPCC 2018). From those estimates we know that many of these effects of Climate Change are much more likely than a Pandemic. Perhaps happening more slowly, giving us more time to respond. As with the models predicting the likely course of the pandemic, we have sufficient information now to model and anticipate how climate change will continue to unfold. We could make use of this information to inform individual and collective actions that will have consequential results for the lethality of climate change.

Those actions require that we build human capacities and the motivation and the skill to affect those individual and collective changes. Climate change education is a field of scholarship and practice that can guide the efforts to build those capacities. Will we be able to act more swiftly and more effectively than we have been to in the case of the pandemic? Will the pandemic have made sufficiently visible for us our footprint on the environment, to cause us to want to slow down climate change? Will we be able to generalize what we have learned about the devastating consequences of a high impact global calamity like the Pandemic and about the consequences of acting effectively or of failing to act to the predicted high impact of Climate Change?

My hope is that university students, and faculty, will see that addressing these questions is core to their mission even, and perhaps especially, as we ponder how this pandemic will change us all and our way of life and how we gear up to build back better in the Pandemic’s aftermath.

## References

[CR1] Blickle, C. (2020, May). *Pandemics change cities: Municipal spending and voter extremism in Germany, 1918–1933*. Report No. 921. New York: Federal Reserve Bank of New York. https://www.newyorkfed.org/research/staff_reports/sr921

[CR2] Brownlee MTJ, Powell RB, Hallo JC (2013). A review of the foundational processes that influence beliefs in climate change: Opportunities for environmental education research. Environmental Education Research.

[CR3] Gibb N (2016). Getting climate-ready: A guide for schools on climate action.

[CR4] Gough N (2002). Ignorance in environmental education research. Australian Journal of Environmental Education.

[CR5] IPCC. (2018). Summary for policymakers. In V. Masson-Delmotte, P. Zhai, H.-O. Pörtner, D. Roberts, J. Skea, P. R. Shukla, A. Pirani, W. Moufouma-Okia, C. Péan, R. Pidcock, S. Connors, J. B. R. Matthews, Y. Chen, X. Zhou, M. I. Gomis, E. Lonnoy, T. Maycock, M. Tignor, & T. Waterfield (Eds.), *Global warming of 1.5°C*. An IPCC special report on the impacts of global warming of 1.5°C above pre-industrial levels and related global greenhouse gas emission pathways, in the context of strengthening the global response to the threat of climate change, sustainable development, and efforts to eradicate poverty. In Press.

[CR6] Johns Hopkins University. (2020). Coronavirus Resource Center. Mortality Analyses. https://coronavirus.jhu.edu/data/mortality. Accessed 11 May 2020.

[CR7] Jorgenson SN, Stephens JC, White B (2019). Environmental education in transition: A critical review of recent research on climate change and energy education. The Journal of Environmental Education.

[CR8] Ojala, M. (2012, October). Hope and climate change: The importance of hope for environmental engagement among young people. *Environmental Education Research, 18*(5), 625–642.

[CR9] Ojala M (2016). Facing anxiety in climate change education: From therapeutic practice to hopeful transgressive learning. Canadian Journal of Environmental Education.

[CR01] Reimers F (2020). Educating Students to Improve the World.

[CR10] Reimers F, Schleicher A (2020). A framework to guide an education response to the COVID-19 Pandemic of 2020.

[CR11] Reimers F, Schleicher A (2020). Schooling disrupted, schooling rethought. How the COVID-19 Pandemic is changing education.

[CR12] Rousell D, Cutter-Mackenzie-Knowles A (2020). A systematic review of climate change education: Giving children and young people a ‘voice’ and a ‘hand’ in redressing climate change. Children’s Geographies.

[CR13] Schon D (1983). The reflective practitioner. How professionals think in action.

